# Analysis of gene expression levels and their impact on survival in 31 cancer-types patients identifies novel prognostic markers and suggests unexplored immunotherapy treatment options in a wide range of malignancies

**DOI:** 10.1186/s12967-022-03670-7

**Published:** 2022-10-12

**Authors:** Claudia Giampietri, Francesca Scatozza, Elena Crecca, Virginia Vigiano Benedetti, Pier Giorgio Natali, Antonio Facchiano

**Affiliations:** 1 Department of Anatomy, Histology, Forensic Medicine and Orthopedics, Unit of Human Anatomy, Sapienza University of Rome, Rome, Italy; 2 Laboratory of Molecular Oncology, Istituto Dermopatico Dell’Immacolata, IDI-IRCCS, Via Monti di Creta, 00167 Rome, Italy; 3 Mediterranean Taskforce for Cancer Control (MTCC), Rome, Italy

**Keywords:** Melanoma, Cholangiocarcinoma, Thymoma, Testis cancer, LAG3, TIM3, OX40, GITR, 4-1BB, TIGIT

## Abstract

**Background:**

Immunotherapy has dramatically improved cancer treatment by inhibiting or activating specific cell receptors, thus unleashing the host anti-tumor response. However, the engagement of the three main immune checkpoints so far identified, CTLA4, PD-1 and PD-L1, is effective in a fraction of patients, therefore novel targets must be identified and tested.

**Methods:**

We focused our attention on the following nine highly relevant immune checkpoint (ICR) receptors: CTLA4, PD1, PD-L1, LAG3, TIM3, OX40, GITR, 4-1BB and TIGIT. All of them are targets of existing drugs currently under clinical scrutiny in several malignancies. Their expression levels were evaluated in patient tissues of 31 different cancer types vs. proper controls, in a total of 15,038 individuals. This analysis was carried out by interrogating public databases available on GEPIA2 portal and UALCAN portal. By the Principal Component Analysis (PCA) their ability to effectively discriminate patients form controls was then investigated. Expression of the nine ICRs was also related to overall survival in 31 cancer types and expressed as Hazard Ratio, on the GEPIA2 portal and validated, for melanoma patients, in patients-datasets available on PROGgene V2 portal.

**Results:**

Significant differential expression was observed for each ICR molecule in many cancer types. A 7-molecules profile was found to specifically discriminate melanoma patients from controls, while two different 6-molecules profiles discriminate pancreatic cancer patients and Testicular Germ Cell Tumors from matched controls. Highly significant survival improvement was found to be related to the expression levels of all nine ICRs in a wide spectrum of malignancies. For melanoma analysis, the relation with survival observed in TCGA datasets was validated in independent GSE melanoma datasets.

**Conclusion:**

Analysis the nine ICR molecules demonstrates that their expression patterns may be considered as markers of disease and strong survival predictors in a variety of malignancies frequently associated to poor prognosis. Thus, the present findings are strongly advocating that exploratory clinical trials are worth to be performed, using available drugs, targeting these molecules.

**Supplementary Information:**

The online version contains supplementary material available at 10.1186/s12967-022-03670-7.

## Background

Developing immunotherapy treatments based on the activation or inhibition of molecules that orchestrate the host immune response is opening new horizons in the treatment options. Indeed, inhibitory- or agonistic- drugs targeting these molecules are currently under common use or scrutiny in several clinical trials with impressive results in different cancer types [1–4]) and have substantially modified survival in an increasing fraction of patients bearing different tumor types, with less severe side effects [5–7]. The most important inhibitory targets so far identified are CTL4 [8, 9], PD-1 and its ligand PD-L1 (CD-274) [10, 11]; many other co-inhibitory molecules were identified such as Adenosine A2A receptors [12], B7-H3 (CD-276) [13], B7-H4 (VTCN1) [14], LAG3, TIM3, and TIGIT [15, 16], as well as co-stimulatory molecules such as CD27 [17], CD28 [18], CD40 [19], OX40 (TNFRSF4), GITR (TNFRSF18), 41BB (TNFRSF9) [20–22]. Despite the remarkable results obtained so far, new targets and new drugs need to be developed and tested, since many patients do not respond to the treatments or even developed resistance in some cases.

One way to identify cancer markers or potential therapeutic targets is to analyze the expression level of potential targets in the blood as well in tumors vs. normal tissue counterparts*.* By using this approach, the aim of the present study was to identify new prognostic markers able to predict response to therapy, or new potential targets of currently available treatments. Since all molecules under investigation in the present study are either targets of existing drugs, or under clinical investigation, the results collected in the present study may be of timely translational relevance. Based on our previous experience in the field of melanoma markers- and therapeutic targets identification [23–32], in the present study we selected nine molecules referred to as immune checkpoint related (ICR) molecules (namely: inhibitory targets such as CTLA4, PD-1, PD-L1, LAG3, TIM3, and TIGIT, and co-stimulatory molecules such as OX40 (TNFRSF4), GITR (TNFRSF18), 4-1BB (TNFRSF9). All such ICRs are relevant players in the immune-response control and are all targets of existing drugs under clinical use or clinical investigation in several cancers. The gene expression levels of the ICR were then evaluated in cancer vs. control patients, in 31 different cancer types, in a total of 15,038 individuals, and relation to diseases state and patient survival was investigated.

## Methods

The specific aim of the current study was to select novel molecular targets showing, on the base of their gene-expression levels, a statistically significant ability to discriminate patients from healthy controls and to predict prognosis and response to therapy. Expression data and survival data analysis were carried out on public portals for cancer patient data, namely GEPIA2 portal (at http://gepia2.cancer-pku.cn/#index), PROGgene V2 portal (at http://www.progtools.net/gene/index.php), UALCAN portal (at http://ualcan.path.uab.edu/analysis.html) and EBI expression atlas at https://www.ebi.ac.uk/gxa/home.

The list of 31 different cancer types under investigation is reported in Table 1, indicating for each cancer type: the full name, the acronym, and the patients-sample numerosity. Cancer types were the ones investigated in GEPIA2 cancer portal which refers to TCGA datasets and GTEx datasets.

**Table 1:** The list of 31 different cancer types under investigation, indicating the acronyms and the sample numerosity for each cancer type and for each corresponding control

Acronym	Cancer type	N. of tumor samples	N. of normal samples
ACC	Adrenocortical carcinoma	77	128
BLCA	Bladder Urothelial Carcinoma	404	28
BRCA	Breast invasive carcinoma	1085	291
CESC	Cervical squamous cell carcinoma and endocervical adenocarcinoma	306	13
CHOL	Cholangiocarcinoma	36	9
COAD	Colon adenocarcinoma	275	349
DLBC	Lymphoid Neoplasm Diffuse Large B-cell Lymphoma	47	337
ESCA	Esophageal carcinoma	182	286
GBM	Glioblastoma multiforme	163	207
HNSC	Head and Neck squamous cell carcinoma	519	44
KICH	Kidney Chromophobe	66	53
KIRC	Kidney renal clear cell carcinoma	523	100
KIRP	Kidney renal papillary cell carcinoma	286	60
LAML	Acute Myeloid Leukemia	173	70
LGG	Brain Lower Grade Glioma	518	207
LIHC	Liver hepatocellular carcinoma	369	160
LUAD	Lung adenocarcinoma	483	347
LUSC	Lung squamous cell carcinoma	486	338
OV	Ovarian serous cystadenocarcinoma	426	88
PAAD	Pancreatic adenocarcinoma	179	171
PCPG	Pheochromocytoma and Paraganglioma	182	3
PRAD	Prostate adenocarcinoma	492	152
READ	Rectum adenocarcinoma	92	318
SARC	Sarcoma	262	2
SKCM	Skin Cutaneous Melanoma	461	558
STAD	Stomach adenocarcinoma	408	211
TGCT	Testicular Germ Cell Tumors	137	165
THCA	Thyroid carcinoma	512	337
THYM	Thymoma	118	339
UCEC	Uterine Corpus Endometrial Carcinoma	174	91
UCS	Uterine Carcinosarcoma	57	78
	Totals	9498	5540

Expression levels of the ICR molecules CTL4, PD-1 (PCD1), PD-L1 (CD274), LAG3, TIM3 (HAVCR2), OX40 (TNFRSF4), GITR (TNFRSF18), 4-1BB (TNFRSF9) and TIGIT were addressed in biopsies of each cancer type vs. the matched healthy tissue, with a significance threshold set at p < 0.0001, according to the tool: Expression analysis / Expression DIY/ Box plot, in GEPIA2 portal.

The principal component analysis (PCA) was carried out at the GEPIA2 portal according to the tool “Dimensionality reduction”, retrieving the 2D plots. Expression of the ICR molecules in the metastatic phase vs. primary tumor, in melanoma patients, was investigated in the cancer portal UALCAN (at http://ualcan.path.uab.edu/analysis.html).

Survival analysis was carried out at GEPIA2 portal using the tool: “Survival analysis”, on Overall Survival data and Median expression, retrieving Hazar Ration (HR) with 95% confidence interval.

Validation of expression data in melanoma human samples for the nine ICRs was achieved by accessing data on the EBI Expression Atlas at https://www.ebi.ac.uk/gxa/home.

Validation of survival data in melanoma patients was achieved on GSE datasets, (i.e. datasets independent from the TCGA datasets used by GEPIA2), at the PROGgene V2 portal (http://www.progtools.net/gene/index.php).

Statistical analyses implemented within the GEPIA2, PROGgene V2 and UALCAN portals were exploited. Significance threshold of p < 0.0001 was chosen.

## Results

### Expression levels of nine immune checkpoint-related (ICR) molecules in 31 cancer types

In the present study we addressed the role Immune Checkpoint-Related (ICR) molecules, either as markers of disease or as markers of response to therapy/therapeutic targets, in 31 cancer types listed in Table 1. This analysis was carried out on expression data derived from the public cancer portal GEPIA2 (http://gepia2.cancer-pku.cn/), which refers to TCGA and GTEx datasets. Namely, CTL4, PD-1 (PCD1), PD-L1 (CD274), LAG3, TIM3 (HAVCR2), OX40 (TNFRSF4), GITR (TNFRSF18), 4-1BB (TNFRSF9) and TIGIT were investigated. Selection of these nine ICR molecules was based on a preliminary analysis, indicating that the selected ICR molecules are all currently investigated in clinical trials involving cancer and non-cancer patients (see Table 1 not shown).

**Fig. 1:**
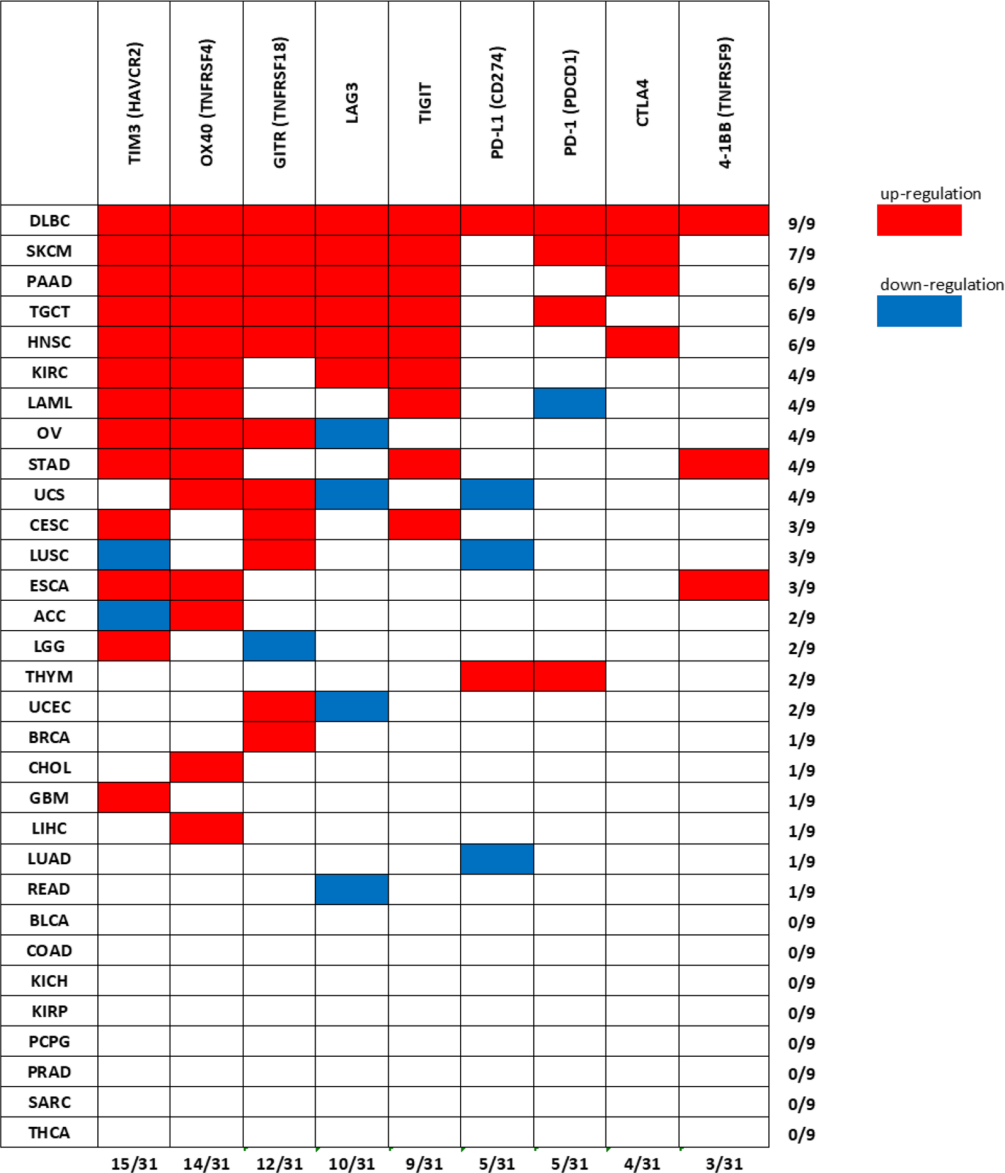
Immune-check point molecules differently expressed in different cancer-types. Red spots indicate significant up-regulation; blue spots indicate significant down-regulation

**Fig. 2:**
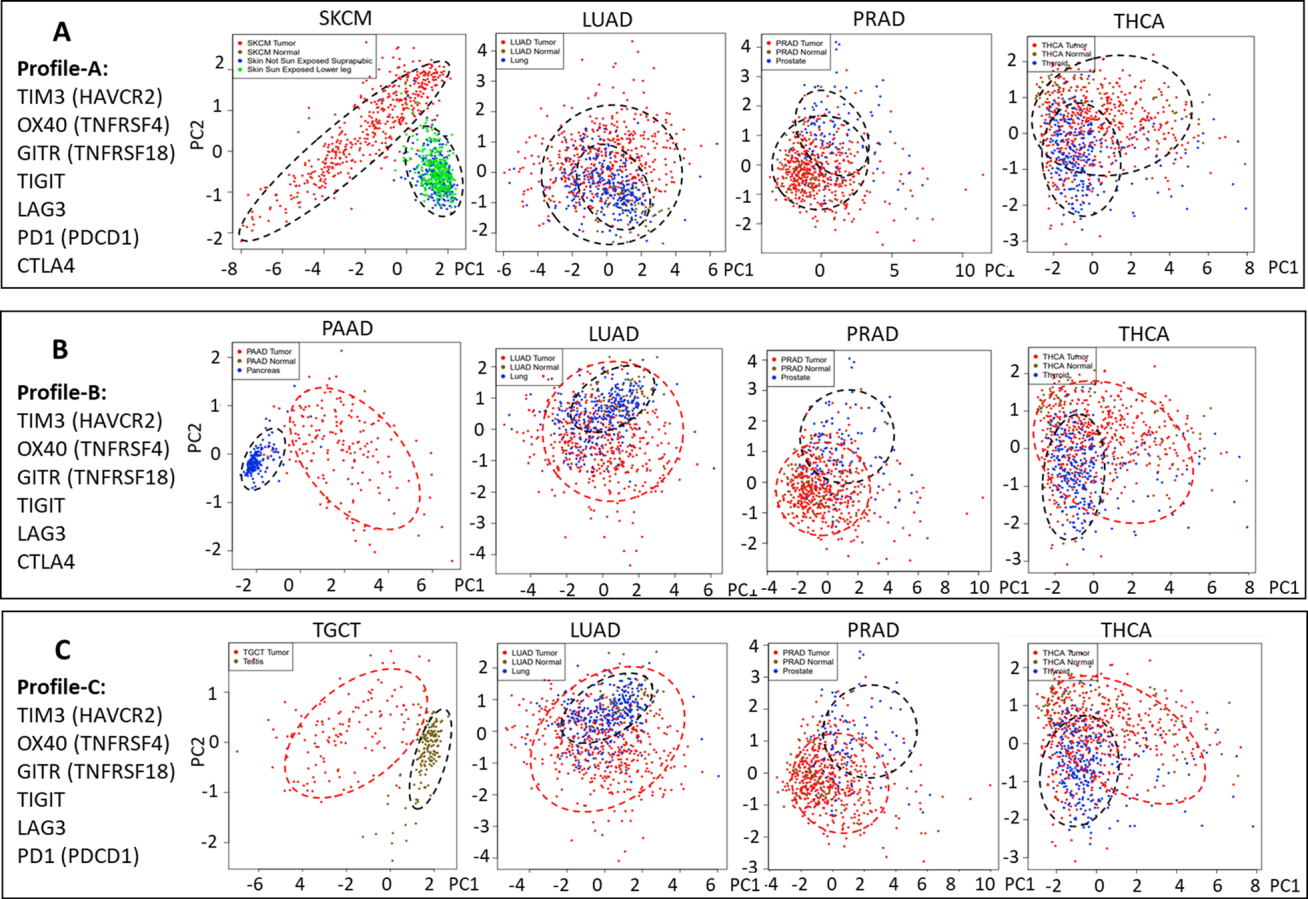
Principal Component Analysis of the Profile-A, Profile-B and Profile-C in SKCM, PAAD and TGCT cancers, respectively. The 3 profiles effectively discriminate patients from controls, in SKCM, PAAD and TGCT, respectively, while they are ineffective in LUAD, PRAD and THCA cancers

Additional file 1: Figures S1 to S9 show expression levels of the ICR molecules in 31 cancer types vs. the corresponding controls, in 9498 cancer samples and 5540 matched-healthy controls. Numerosity of cancer samples and corresponding controls for each cancer type is reported in Table 1. Boxes in Additional file 1: Figures S1 to S9 highlight the cases where a significantly different expression (p < 0.0001) is found in the given cancer vs. the corresponding controls. Data reported in Additional file 1: Figures S1 to S9 are summarized in Fig. 1 reporting the expression data in a heatmap modality. Cancer types are sorted vertically from the top (higher number of significant differences observed) to the bottom (lower number of significant differences observed). ICR molecules are sorted horizontally from the left (higher number of significant differences found) to the right (lower number of significant differences). Red spots indicate significant up-regulation and blue spots indicate significant down-regulation. Figure 1 shows that Lymphoid Neoplasm Diffuse Large B-cell Lymphoma (DLBC) is at the top first position since DLBC patients show expression of all nine molecules up-regulated in cancer vs. controls. Skin Cutaneous Melanoma (SKCM) is at the second top position, since SKCM patients show 7 out 9 molecules up-regulated. Such 7 molecules represent the “Profile-A” investigated later in Fig. 2A. Pancreatic Adenocarcinoma (PAAD) and Testicular Germ Cell Tumors (TGCT) are at the third and fourth top position, since PAAD and TGCT patients show 6 out of 9 molecules up-regulated. Such two 6-molecules combinations are the “Profile-B” and “Profile-C”, investigated later in Fig. 2B, C. Figure 1 then reports all other cancer types showing 5, 4, 3, 2, 1 and 0 ICR molecules differently expressed, according to the vertical sort. It is noteworthy that 23 cancer types out of 31 show at least 1 significantly different expression. ICRs expression data reported in Fig. 1 and Additional file 1: Figures S1 to S9 were from TCGA datasets analyzed in GEPIA2. Expression data of ICRs in melanoma, in pancreatic adenocarcinoma and in thyroid carcinoma were validated in the EBI expression atlas, from independent datasets, as reported in Additional file 1: Table S3 not shown, Additional file 1: Table S4 not shown and Additional file 1: Table S5 not shown, respectively.

Figure 1 also shows that TIM3 (HAVCR2) and OX40 (TNFRSF4) are at the most left positions; they are differently expressed in 15 and 14 cancers types respectively, out of 31, followed by GITR (TNFRSF18), LAG3 and TIGIT, differently expressed in 12 and 10 and 9 cancers, respectively. The other ICR molecules are then reported according to the left-to-right horizontal sort.

The large number of significant differences reported in Fig. 1 indicate that such ICR molecules may have a potential role in several cancer types, either as diagnostic/prognostic markers or as therapeutic targets. We then hypothesized that ICRs expression may represent a profile able to effectively discriminate cancer samples from the corresponding healthy controls. This hypothesis was verified using the Dimensionality Reduction tool of GEPIA2, which performs a PCA analysis on the combined expression data. PCA analysis of all nine ICRs in Lymphoid Neoplasm Diffuse Large B-cell Lymphoma patients (DLBC) was not possible, due to the lack of a proper control dataset to be used in PCA calculation. However, PCA analysis of the “Profile-A” was possible in melanoma (SKCM) patients. In fact, Fig. 2A shows that the 7 molecules differently expressed in SKCM samples (i.e., TIM3 (HAVCR2), OX40 (TNFRSF4), GITR (TNFRRSF18), TIGIT, LAG3, PD-1 (PDCD1) and CTL4), show a clear separation of patients from the corresponding controls. Figure 2A also shows that Profile-A is not able to discriminate LUAD, PRAD and THCA cancer patients from the corresponding controls, indicating that such profile appears to be specific for melanoma patients (SKCM).

“Profile-B” is made by the 6 molecules differently expressed in Pancreatic adenocarcinoma (PAAD) patients (i.e., TIM3 (HAVCR2), OX40 (TNFRSF4), GITR (TNFRRSF18), TIGIT, LAG3 and CTLA4). PCA analysis of Profile-B expression shows a clear separation of PAAD patients form the corresponding controls (Fig. 2B), while the same profile is not able to discriminate LUAD, PRAD and THCA cancer patients from the corresponding controls, indicating that such profile appears to be specific for PAAD patients. Similarly, when the “Profile-C” expression (i.e., TIM3 (HAVCR2), OX40 (TNFRSF4), GITR (TNFRSF18), TIGIT, LAG3 and PDCD1) was investigated in Testicular Germ Cells Tumor (TGCT) samples, a clear separation from the corresponding controls was found (Fig. 2C), while the same profile does not discriminate LUAD, PRAD and THCA cancer patients from the corresponding controls, indicating that such profile appears to be specific for TGCT patients. Figure 2 shows that the 7 molecules differently expressed in SKCM, and the six molecules differently expressed in PAAD and in TGCT are specific expression-profiles able to clearly discriminate cancers form controls samples, according to PCA analysis, showing a relevant marker potential for specific cancer types.

**Fig. 3:**
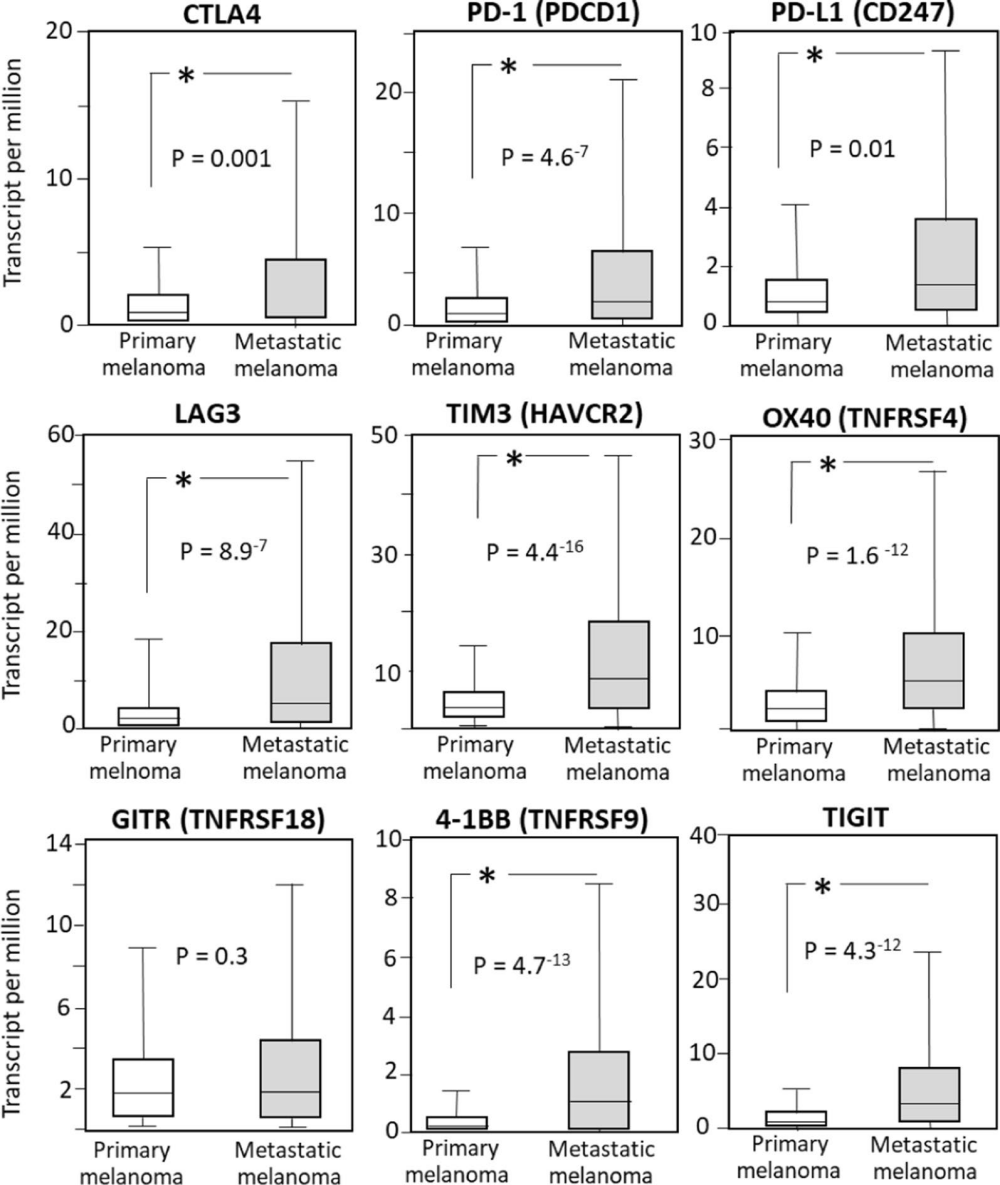
Gene expression levels of the 9 ICRs in primary (N = 104) and metastatic (N = 368) melanoma patients

We then investigated the role the nine ICR molecules may play in the metastatic phase of melanoma, by accessing the expression data in 104 melanoma primary patients vs. 368 melanoma metastatic patients, at the UALCAN public portal (http://ualcan.path.uab.edu/analysis.html). All ICRs investigated, except GITR (TNFRSF18), show a significantly different expression in primary vs. metastatic patients, as reported in Fig. 3, suggesting that such molecules (or drugs targeting these molecules) may have a role in metastatic melanoma diagnosis or control.

### Patients survival analysis

We then addressed the hypothesis that the nine ICRs may represent potential suitable therapeutic targets or markers effectively predicting prognosis or response to therapy. We investigated such hypothesis by analyzing how their expression relates to patient survival. This analysis may indicate molecules with a potential therapeutic application, irrespective of their marker ability to identify cancer patients. Table 2 reports survival data expressed as Hazard Ratio (HR) as a function of expression values of each ICR molecules, according to GEPIA2 database. Expression levels were categorized as “high-level” (i.e., above the median value) and “low-level” (i.e., below the median value). In Table 2 only HR ≤ 0.7 or ≥ 1.5 are reported, where HR < 1 indicates improved survival in high-level expressing patients, while HR > 1 indicates improved survival in low-level expressing patients. Data in Table 2 are both vertically and horizontally sorted, similarly to Fig. 1. Surprisingly, horizontal sorting shows that in Skin Cancer Melanoma (SKCM), in Testicular Germ Cell Tumors (TGCT) and in Thymoma (THYM) the expression of all nine ICR molecules is associated with relevant survival improvement. In TGCT and THYM, survival improvement is almost invariantly associated with low-expression levels of all ICRs except PD-1 (PDCD1), while improved survival in SKCM is always associated with high expression levels of all nine molecules. Notably, SKCM and TGCT are in the very top positions both in Fig. 1 (expression data) and in Table 2 (survival data).

**Table 2:** Survival Hazard Ratio (HR) calculated according to expression values of 9 ICR molecules (only HR ≤ 0.7 ≥ 1.5 are reported)

	Cancer type	OX40 (TNFRSF4)	TIM3 (HAVCR2)	PD-1 (PDCD1)	4-1BB (TNFRSF9)	TIGIT	LAG3	GITR (TNFRSF18)	CTLA4	PD-L1 (CD274)	Horizontal Summary
1	SKCM	0.59	0.54	0.57	0.59	0.56	0.52	0.58	0.63	0.56	9/9
2	TGCT	3.1	> 10	2	> 10	2	2	4.2	> 10	> 10	9/9
3	THYM	2.9	3	0.47	1.5	> 10	6.1	2.2	5	7.6	9/9
4	LGG	1.7	1.7	1.9	1.5		1.6		1.8	1.8	7/9
5	KIRC		0.68				1.5	1.9	1.5	0.62	5/9
6	READ			0.58	0.63	0.67		0.68	0.61		5/9
7	UCEC		0.44	0.43	0.47	0.5			0.62		5/9
8	ACC	1.5	0.52			0.56	2.2		0.65		5/9
9	DLBC		0.52	0.6	0.69					1.6	4/9
10	KICH	1.6	0.34	0.69				0.48			4/9
11	PCPG	0.58			2.8			0.17	0.48		4/9
12	KIRP	2.1	0.49		2.5		1.7				4/9
13	BRCA			0.65		0.66		0.67			3/9
14	CESC	0.61	0.67			0.53					3/9
15	CHOL		0.47		0.53			0.64			3/9
16	LAML			2.2			1.8			1.7	3/9
17	PRAD	0.53				0.39				0.62	3/9
18	HNSC	0.58				0.63					2/9
19	THCA	1.6		0.5							2/9
20	ESCA						1.5				1/9
21	GBM				1.5						1/9
22	LIHC	1.6									1/9
23	PAAD	0.59									1/9
24	SARC			0.62							1/9
25	UCS		0.67								1/9
26	BLCA										0/9
27	COAD										0/9
28	LUAD										0/9
29	LUSC										0/9
30	OV										0/9
31	STAD										0/9
	Vertical summary	14/31	13/31	12/31	11/31	10/31	9/31	9/31	9/31	8/31	

On the other hand, Table 2 shows that the highest number of cancer types with improved survival associates with expression levels of TIM3 and OX40 (14 out of 31 cancer types and 13 out of 31 cancer types, respectively). Most interestingly, TIM3, OX40 and TIGIT are in the very left positions both in expression and in survival analyses (Fig. 1 and Table 3, respectively).

**Table 3:** Survival in melanoma patients, according to the expression values, in melanoma GSE datasets (http://www.progtools.net/gene/index.php)

Gene	GSE*	HR (Hazard Ratio)	P value	Median survival in high express patients (days)	Median survival in low express patients (days)	Survival at 5 years in high express patients (%)	Survival at 5 years in low express patients (%)
CTLA4	19,234	0.8	*0.5*	3943	1096	61	50
PD-1 (PDCD1)	19,234	0.09	*0.02*	3943	1397	63	46
PD-L1 (CD274)	53,118	0.3	*0.3*	5481	3005	76	52
LAG3	19,234	0.35	*0.009*	4821	955	67	42
TIM3 (HAVCR2)	53,118	0.74	*0.04*	6899	3271	68	58
OX40 (TNFRSF4)	19,234	0.45	*0.1*	3943	2336	62	48
GITR (TNFRSF18)	53,118	0.19	*0.05*	6899	1734	81	46
TIGIT	19,234	0.45	*0.01*	NA**	817	71	40
4-1BB (TNFRSF9)	53,118	0.3	*0.006*	6899	3005	73	55

^*^When data were available from more than one GSE, the one showing the highest difference in median survival is reported

**Fig. 4:**
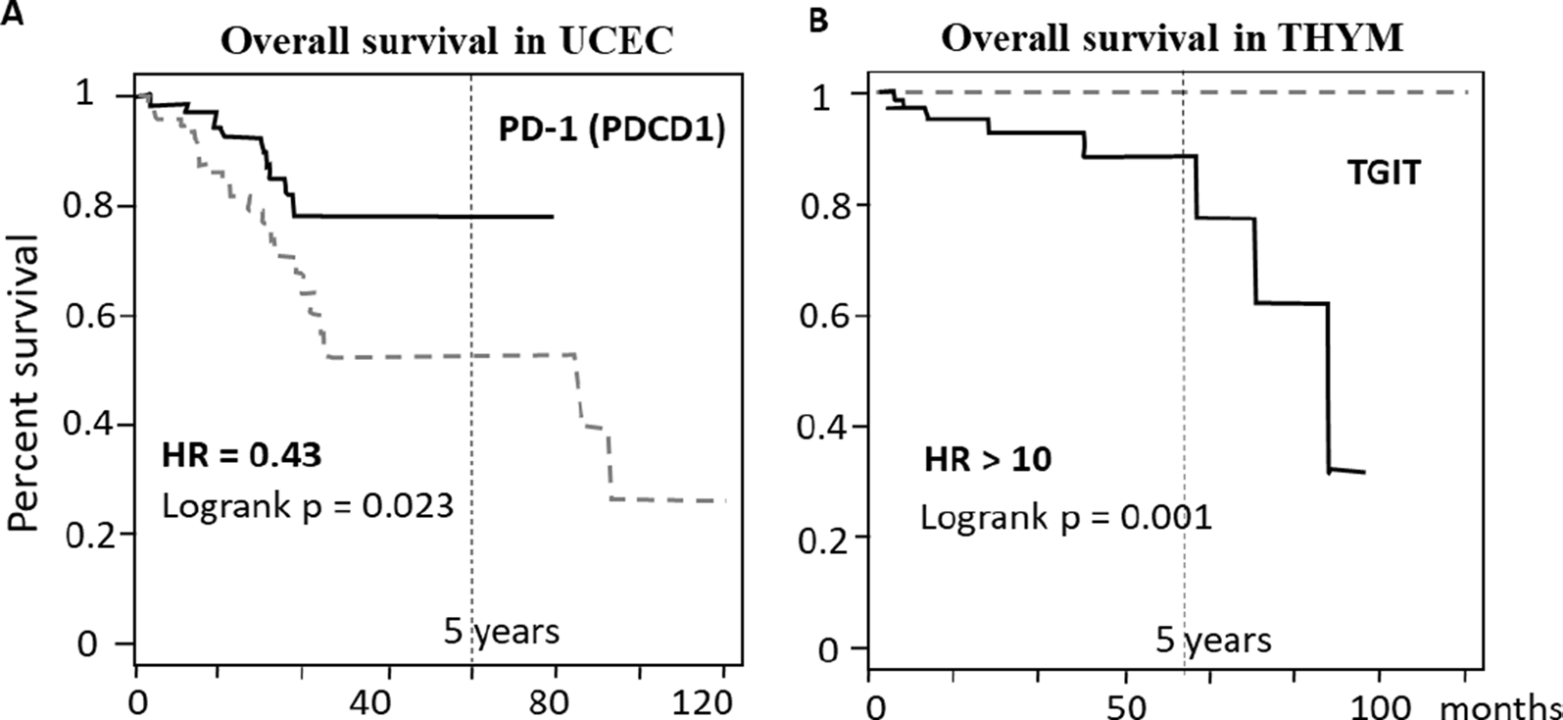
Kaplan–Meier plots indicating survival in UCEC patients (**A**) and in THYM patients (**B**) associated with high- and low-expression levels of PD-1 and TIGIT, respectively. Continuous lines indicate high-level expressing patients; dashed lines indicate low-level expressing patients

Table 2 shows that in several cases HR was found to be smaller than 0.5 or largely higher than 1.5, suggesting a very strong association of the expression to the survival, as for instance for PD-1 (PDCD1) expression in Uterine Corpus Endometrial Carcinoma (UCEC) (see the corresponding Kaplan–Meier plots in Fig. 4A), or for TIGIT expression in Thymoma (THYM) (see the corresponding Kaplan–Meier plots in Fig. 4B). Interestingly, Fig. 4 shows a striking complete survival in patients expressing low-levels of TIGIT as compared to high-levels expressing patients showing a time-dependent loss of survival. Such data indicate that drugs inhibiting TIGIT may exert a potential strong protective role (Fig. 4B) and that PD-1 agonists may have an impact on UCEC patients-survival (Fig. 4A).

Data reported above indicate that expression of several molecules associate with strong survival improvement in melanoma, in Testicular Germ Cell Tumors, in Thymoma as well as in other cancer types, introducing the hypothesis that targeting such molecules may represent an effective therapeutic option in different cancer types.

### Validation of survival data

**Fig. 5:**
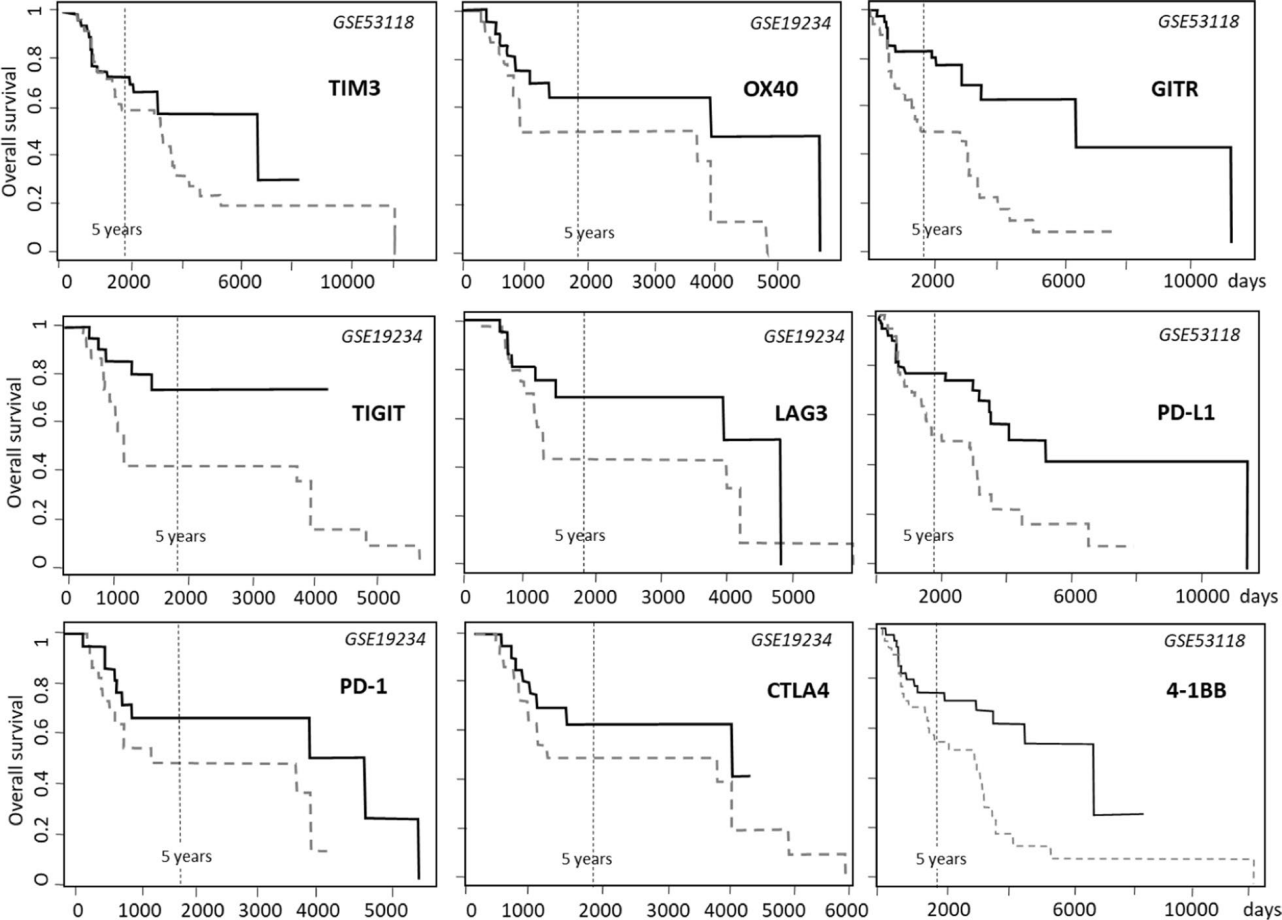
Kaplan–Meier plots indicating survival in melanoma patients according to data reported in PROGgene V2 portal (http://www.progtools.net/gene/index.php). Continuous lines indicate high-level expressing patients; dashed lines indicate low-level expressing patients

The nine ICRs showing a relevant survival association in melanoma patients according to data reported in TCGA datasets (Table 2), were all validated by analyzing independent GSE datasets, at the PROGgene V2 portal (http://www.progtools.net/gene/index.php). Figure 5 reports the corresponding Kaplan–Meier survival plots and Table 3 reports the corresponding numerical analysis.

Validation achieved on GSE datasets confirm the interest of all such molecules as potential therapeutic targets in melanoma patients.

## Discussion

Immunotherapy represents one of the most important advancements in the cancer therapy field, since inhibitors of immune checkpoints CTLA4, PD-1 and PD-L1 dramatically improved therapeutic results [33]. However, as underlined [34], despite the strong positive impact of these drugs in many patients, many others do not receive relevant advantages, pointing toward the need to identify additional targets and drugs. Furthermore, as reported in Table 2 not shown, the Clinicaltrials.gov portal reports several “ongoing” or “completed” immunotherapy-based clinical trials addressing melanoma, lung cancers, breast cancers, and renal cancers, while only a few clinical immunotherapy-based trials are reported for other cancers such as testis cancers, adrenocortical cancers, thymoma, pheochromocytoma and paraganglioma, and thyroid cancers. Cancers response to immunotherapy has been shown to be related to their immune landscape, i.e., “active”, or “deserted” or “excluded” immune landscape [35] or to a simpler two-levels landscape, i.e., immune-active and immune-silent background [36]. The immune landscape relates to the balance between immune-activating and immune-suppressing mechanisms, ultimately controlling tumor-escape, as recently investigated in cancers undergoing anti-CTLA4 and anti PD-1 antibodies treatment [37]. The presence of the immune-active or -silent environment is related to five mechanisms (i.e. tissue-specific destruction, autoimmunity, tumor rejection, pathogen clearance, allograft rejection) requiring the coordinated involvement of three major players, i.e., T cell response, self- non-self-recognition and innate immunity [38]. Indeed, nineteen upstream molecular mechanisms and signatures control the way cancers comply or escape to the immune response, including WNT/βCatenin, MAPK, Immunogenic Cell Death, Regulatory T cells, IL23-Th17 axis, PI3K signature, SHC1 signature, IDO/NOS signature and others [36].

Besides CTLA4, PD-1 and PD-L1, several additional immune checkpoints related (ICR) molecules have been identified such as LAG3, TIM3 (HAVCR2), OX40 (TNFRSF4), GITR (TNFRSF18), 4-1BB (TNFRSF9), TIGIT, currently under active clinical investigation. The present study aimed at obtaining clinical relevant information by addressing whether prognostic correlations exist between expression of ICR molecules and patients’ survival, as also investigated in previous studies [39, 40]. We hypothesized that the nine molecules investigated, while being immune-check point related molecules all strongly related to each other, may be also involved in many different functions which coordinately converge toward the control of cancer growth. This hypothesis was supported by a preliminary interactome analysis with STRING (at https://string-db.org/), which indicated functional/molecular interactions of the ICRs with the previously mentioned models controlling immune response to cancers [36] and with the signature underlying the Immunologic Constant of Rejection, which controls innate and adaptative immune effectors-mediated tissue-specific destruction, autoimmunity, pathogen-bearing cells clearance, acute allograft rejection, and rejection of cancer [36, 38]. Namely, STRING analysis found functional/molecular interactions between CTLA4 and βCatenin, between PD-L1 and βCatenin, between CTLA4 and IL23, between PD-L1 and SHC1, between PD-L1 and PI3K, between OX40 and NOS, between GITR and NOS, thus suggesting some nodes functionally linking ICRs to the Immunologic Constant of Rejection.

The novelty of this study is related to the strong translational potential of the results, supported by: (a) the nine molecules investigated are all targets of drugs already under clinical evaluation; (b) a large representative number of samples (15,038 individuals) was studied; (c) the screening phase performed on TCGA datasets, was paralleled, when possible, by a validation phase in independent cancer datasets; (d) a more in-depth validation was performed in melanoma. As a possible limitation, the current analysis does not consider prognostic factors such as age or gender or tumor-stage, which refer to data unavailable in the GEPIA2 portal. However, analyses here reported provide clinically informative results. Namely, gene expression level of all nine ICRs is significantly changed in Lymphoid Neoplasm Diffuse Large B-cell Lymphoma (DLBC) patients; expression of 7 out of 9 ICRs is significantly modified in melanoma (SKCM) while expression of 6 out of 9 is altered in Pancreatic adenocarcinoma (PAAD) and in Testicular Germ Cell Tumors TGCT patients. Such data (reported in Fig. 1 and Additional file 1: Figures S1 to S9), suggest that these molecules may represent markers profiles (Profile-A, Profile-B and Profile-C), able to effectively discriminate patients from controls. This hypothesis was supported by PCA analysis reported in Fig. 2, indicating the actual ability of the 3 profiles to discriminate SKCM, PAAD and TGCT from controls, respectively, as well as their in-ability to discriminate LUAD, PAAD and THCA cancers patients from controls. Further, according to Fig. 3, expression levels of all nine ICRs (except GITR) may also represent a hallmark of metastatic phase in melanoma patients.

Analyses evaluating the overall survival, based on the gene expression values in patients irrespective of the expression values in controls, identified potential therapeutic targets or markers able to predict response to therapy. Survival analysis revealed relevant data in several cancer types and were validated in melanoma patients in independents datasets (see Fig. 5).

ICRs showing the highest survival impact in several cancer types are individually discussed below.

CTLA4 (Cytotoxic T-Lymphocyte Associated Protein 4) is one of the most important negative regulators of T cells and one of the main inhibitory immune checkpoints. It is the target of a few drugs such as ipilimumab and tremelimumab. Clinical indications of CTLA4 inhibitors, often in combination with PD-1/PD-L1 inhibitors, include several advanced or metastatic cancers [41, 42]. Survival data reported in Table 3 of the current study indicate that low CTLA4 expression levels associate with strong survival in TCGT and in THYM patients (HR > 10 and HR = 5, respectively), thus suggesting that CTLA4 inhibitors may have beneficial effects in these malignancies. To date (June 2022) a very limited number of studies is reported in Clinicaltrials.gov portal, investigating CTLA4 inhibitors in testis cancers or in thymic cancer patients.

PD-1 (PDCD1) (Programmed Cell Death Protein 1) is another key inhibitory receptor expressed on T-cells, inducing immune tolerance. It is the target of antibodies drugs such as nivolumab, pembrolizumab, sintilimab, which reinforce the immune response by inhibiting PD-1. Clinical indications of PD-1 inhibitors include several cancers in advanced stage [43, 44]. Table 2 indicates that low PD-1 (PDCD1) expression levels associate with strong survival improvement in Testicular Germ Cell Tumors (TCGT), Brain Lower Grade Glioma (LGG) and Acute Myeloid Leukemia (LAML), (HR 2, 1.9 and 2.2, respectively), thus suggesting that drugs inhibiting this molecule may be beneficial in such patients. While several studies are reported addressing the effect of PD-1 (PDCD1) inhibitors in gliomas and leukemia patients, to date only one study is reported in Clinicaltrials.gov investigating PD-1 inhibitors (namely nivolumab) in testis cancers [45]. On the other hand, PD-1 (PDCD1) high-levels expression associate with substantially improved survival in a few other cancer types, such as UCEC, as reported in Table 2 and Fig. 2A, therefore we suggest that PD-1 (PDCD1) agonists may have beneficial effects in these patients.

PD-L1 (CD274) (Programmed Cell Death 1 Ligand 1) is another key T cells negative regulator able to induce immune tolerance. PD-L1 inhibiting drugs, such as atezolizumab, durvalumab and avelumab, are currently used in cancer therapy to burst the immune response. Clinical indications of PD-L1 inhibitors include several advanced solid and blood cancers [46]. Table 2 of the current study indicates that low expression levels of PD-L1 (CD274) associate with strong survival improvement in TGCT, THYM, LGG, DLBC and LAML, suggesting that inhibitors of this molecule may have a beneficial effect in these tumors. Consistently with these data, several clinical trials are reported in Clinicaltrials.gov on PD-L1 and glioma, or lymphoma, or leukemia. On the contrary, only a few studies are reported on other cancer types, namely testis cancer and thymoma.

LAG3 (Lymphocyte Activating 3) is a key immune checkpoint molecule negatively controlling immune response in cancer, infectious diseases and autoimmunity [47, 48]. It is the target of different drugs including relatlimab, an anti-LAG3 antibody recently approved by FDA for clinical use in melanoma, in combination with nivolumab. In fact, results of the RELATIVITY-047 study [49] indicate a significant improvement of Progression-Free Survival (PFS) in melanoma patients treated with nivolumab + relatlimab, where the relatlimab combination with anti-PD-1 drugs is a strategy to overcome drug resistance [50]. Table 2 of the present study indicates that low expression levels of LAG3 is associated with favorable survival in several cancer types including TGCT, THYM, KIRC, KIRP, LGG, ACC, LAML and ESCA. This suggests that LAG3 inhibitors may have a therapeutic potential in these cancers. Nevertheless, Clinicaltrials.gov portal reports only a few ongoing studies regarding LAG3 inhibitors in renal and esophageal tumors, in glioma, in leukemia, in testis cancer. No studies at all are reported on LAG3 and thymoma, nor LAG3 and adrenocortical carcinoma.

TIM3 (HAVCR2) (Hepatitis A Virus Cellular Receptor 2) is a key checkpoint molecule inhibiting the innate and adaptive immune response in cancer. It is the target of LY-3321367, an anti-TIM3 (HAVCR2) antibody. It is currently investigated in solid tumors under progression (such as NSCLC, gastric and urothelial carcinoma [51] and it is suggested as a potential therapeutic target in Acute Myeloid Leukemia [52]. Table 2 indicates that TIM3 (HAVCR2) low expression levels associate with improved survival in TGCT, THYM and LGG, thus suggesting the use of TIM3 (HAVCR2) inhibitors in such patients. Nevertheless, only a few studies investigate TIM3 (HAVCR2) inhibitors in glioma, testis cancer, and thymoma. Alternatively, consistently with the reported dual role of TIM3 (HAVCR2) [53], survival data in Table 2 also indicate that high expression levels of TIM3 (HAVCR2) associate with improved survival in 10 cancer types, including SKCM, KIRC, ACC, DLBC, UCEC, CHOL and others. This may suggest that TIM3 (HAVCR2) agonists may have a role in these cancer types. Nevertheless, according to the Clinicaltrials.gov portal, TIM3 (HAVCR2) related drugs are poorly investigated in kidney cancers, in adrenocortical cancer, in uterus cancer and in cholangiocarcinoma.

OX40 (Tumor necrosis factor receptor superfamily, member 4 TNFRSF4) is a costimulatory immune checkpoint molecule. OX40 agonistic monoclonal antibodies recognize OX40, leading to T cell activation. Several OX40 (TNFRSF4) targeting molecules, such as ivuxolimab, vonlerolizumab and tavolimab, show in clinical trials preliminary anti-cancer action in locally advanced or metastatic cancers [54]. According to Table 2 of the present study, OX40 (TNFRSF4) expression levels associate with an improved survival in as many as 14 different cancer types. Improved survival is observed in high-level expressing patients of SKCM, CESC, HNASC, PAAD, PCPG, and PRAD, therefore, OX40 (TNFRSF4) agonists may be useful in such patients. On the contrary, improved survival is observed in low-levels expressing patients of TGCT, THYM, LGG, ACC, KICH, KIRP, THCA and LIHC, suggesting the use of OX40 (TNFRSF4) antagonists in these patients. However, according to clinicaltrials.gov portal, almost no studies are reported for OX40 (TNFRSF4) related molecules in pancreas cancers, prostate cancers, pheochromocytoma cancers, testis cancer, thymoma, glioma, thyroid cancers nor in adrenocortical cancers. Considering the poor prognosis of most of these cancers, these data may represent a valuable indication for future clinical studies.

TIGIT is a T-cell immunoreceptor with Ig and immunoreceptor tyrosine-based inhibitory motif (ITIM), with negative regulatory action on T-cells. The use of a TGIT inhibitor is currently evaluated in glioblastoma, glioma, and other advanced solid tumors [55]. According to Table 2, TIGIT expression levels associate with significantly improved survival in 10 out of 31 cancer types. In most cases, survival is improved in high expression-levels patients, for instance in SKCM, ACC, PRAD, READ, UCEC or BRCA. TIGIT inhibitors may therefore worth to be tested in these patients. However, Clinicaltrials.gov reports very few or no studies on TIGIT targeting drugs in prostate cancers, nor in adrenocortical carcinoma, nor rectum cancer, endometrial cancers, breast cancers. The strong survival improvement associated with TIGIT expression levels (reported in Table 2 and in Fig. 4B) suggests that TIGIT-inhibitors drugs may be clinically beneficial in thymoma and Testicular Germ Cell Tumors, while TIGIT-agonists may improve survival in SKCM, READ, UCEC, ACC, BRCA, CESC, PRAD and HNSC patients. Presently, only 3 clinical trials are reported on TIGIT-related drugs in lymphoma, and no studies relate TIGIT drugs to thymoma nor to testis cancers.

GITR (TNFRSF18) (TNF Receptor Superfamily Member 18) is a key positive regulator of the immune response. Agonists drugs of this molecule, such as TRX-518, are likely to improve immune response against cancers [56]. Survival data of Table 2 indicate cancer types where high-expression levels of GITR associate with strongly improved survival, namely SKCM, KICH, READ, BRCA, CHOL and PCPG. On the other hand, low-expression levels of GITR associate with strongly improved survival in TGCT, THYM and KIRC. Therefore, GITR inhibitors and GITR agonists may have interesting effects in several cancers, while Clinicaltrials.gov portal shows no clinical trials on GITR-related drugs in cholangiocarcinoma, pheochromocytoma, testis cancers nor in thymoma.

4-1BB (TNFRSF9) (TNF Receptor Superfamily Member 9) is a costimulatory molecule, key controller of immune response. It is the target of different activating or inhibitory monoclonal antibodies, such as urelumab and utomilumab, respectively. They are both currently under investigation in blood- as well as solid malignancies. Table 2 indicates an impressive survival improvement related to 4-1BB expression levels, in several cancer types; consistently, several studies are reported on Clinicaltrials.gov, but very few are reported on Glioma/Glioblastoma, and none on testis cancer nor on Thymoma.

**Fig. 6:**
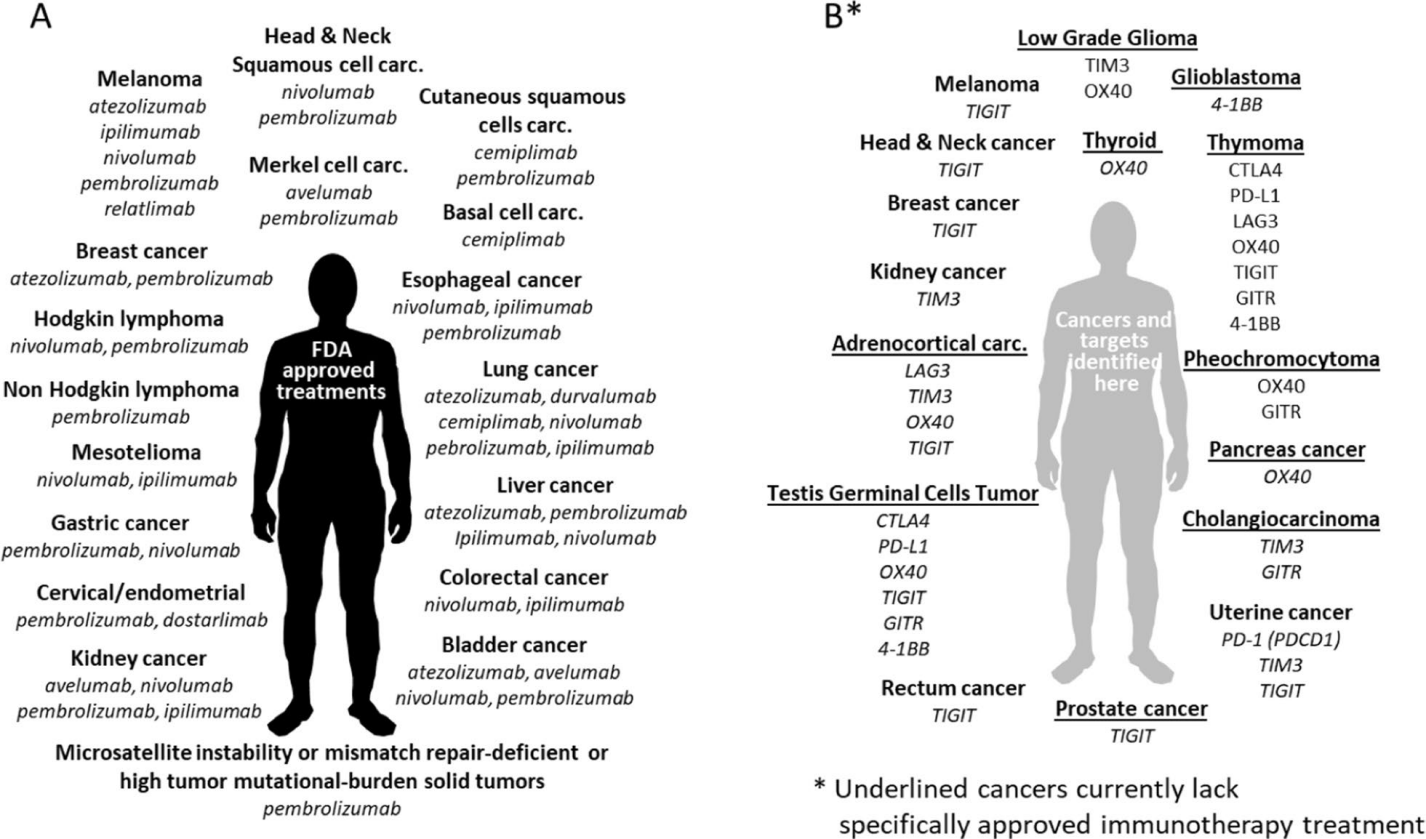
Immunotherapy based treatments approved by FDA (**A**) and new options and molecular targets selected in the present study (**B**)

In conclusion, the expression levels of the nine ICR molecules investigated in the present study show strong and significant differences in several cancers vs. controls, as well as relevant association with overall survival, in several cancer types. In many of these malignancies no or very-few studies are reported regarding drugs targeting these molecules, as for instance in testis cancer, thymoma, gliomas, adrenocortical carcinoma, cholangiocarcinoma. Data reported in the present study indicate potential prognostic markers and may suggest that several drugs, already under clinical use or under clinical evaluation, may have relevance in cancer types such as testis cancer, thymoma, glioma, cholangiocarcinoma, pheochromocytoma, adrenocortical cancer, kidney cancer and uterus cancers. The cartoon reported in Fig. 6 summarizes FDA-approved immunotherapy treatments according to AACR Cancer Progress Report 2021 [57] (Fig. 6A), while cancers and targets selected in the present study are reported in Fig. 6B.

## Supplementary Information

**Additional file 1.** Supplemental material.

## Data Availability

All data analyzed come from public databases, available at links clearly indicated in the manuscript.
