# Supplementary material for: Analysis of gene expression levels and their impact on survival in 31 cancer-types patients identifies novel prognostic markers and suggests unexplored immunotherapy treatment options in a wide range of malignancies
Source: J Transl Med. 2022 Oct 12;20:467. doi: 10.1186/s12967-022-03670-7 (PMC9559014; doi:10.1186/s12967-022-03670-7)
Supplement: Supplementary file 1 — Additional file 1. Supplemental material. [file 12967_2022_3670_MOESM1_ESM.docx]

**Additional material**

**Figure S1.** Gene expression level of CTLA4 in 31 cancer types and in matched healthy controls. Asterisks indicate significant (p < 0.0001) difference.

**Figure S2.** Gene expression level of PD-1 in 31 cancer types and in matched healthy controls. Asterisks indicate significant (p < 0.0001) difference.

**Figure S3.**  Gene expression level of PD-L1 in 31 cancer types and in matched healthy controls. Asterisks indicate significant (p < 0.0001) difference.

**Figure S4.** Gene expression level of LAG3 in 31 cancer types and in matched healthy controls. Asterisks indicate significant (p < 0.0001) difference.

**Figure S5.** Gene expression level of TIM3 in 31 cancer types and in matched healthy controls. Asterisks indicate significant (p < 0.0001) difference.

**Figure S6.** Gene expression level of OX40 in 31 cancer types and in matched healthy controls. Asterisks indicate significant (p < 0.0001) difference.

**Figure S7.** Gene expression level of GITR in 31 cancer types and in matched healthy controls. Asterisks indicate significant (p < 0.0001) difference.

**Figure S8.** Gene expression level of 4-1BB in 31 cancer types and in matched healthy controls. Asterisks indicate significant (p < 0.0001) difference.

**Figure S9.** Gene expression level of TIGIT in 31 cancer types and in matched healthy controls. Asterisks indicate significant (p < 0.0001) difference.

**Figure S1**


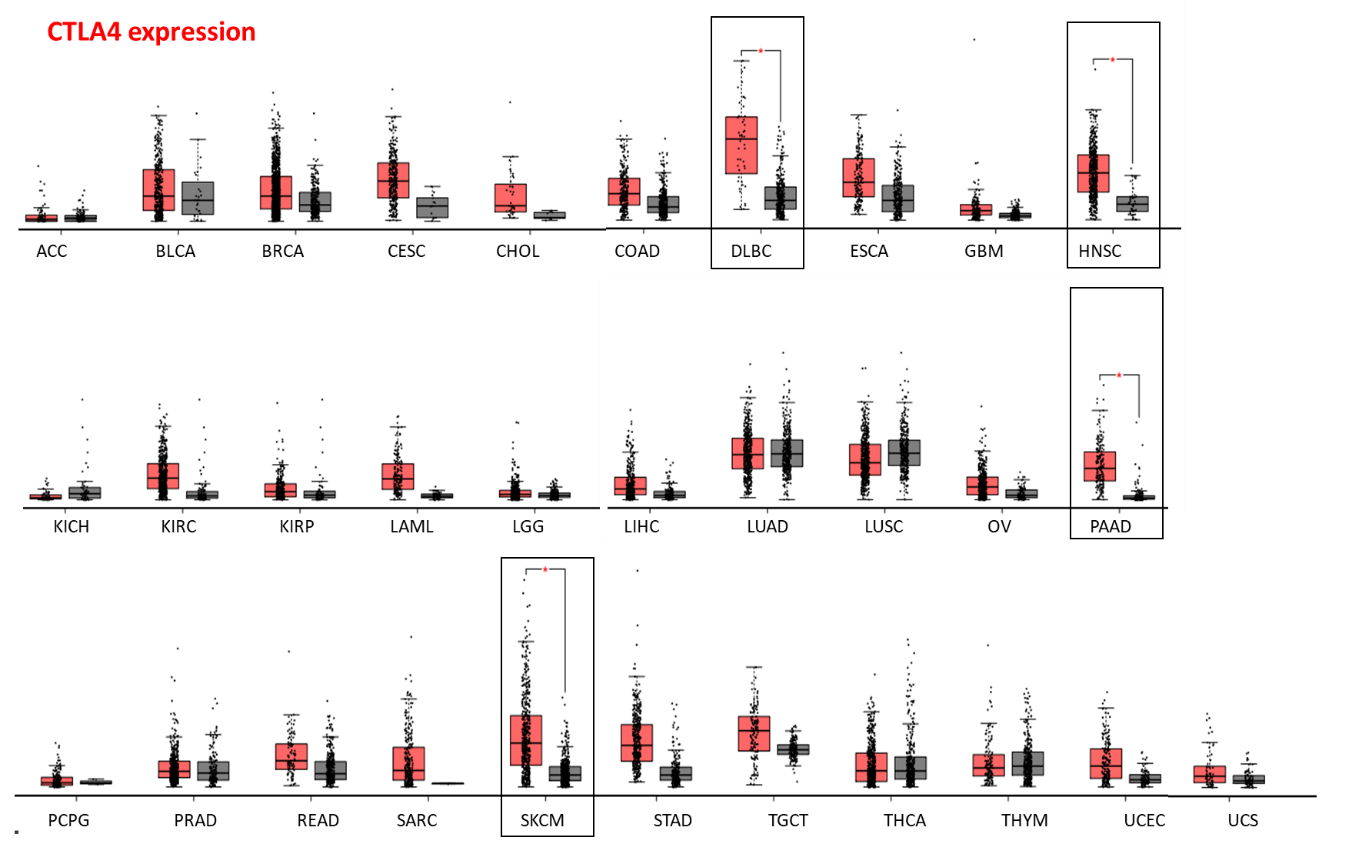


**Figure S1.** Gene expression level of CTLA4 in 31 cancer types and in matched healthy controls. Asterisks indicate significant (p < 0.0001) difference.

**Figure S2**


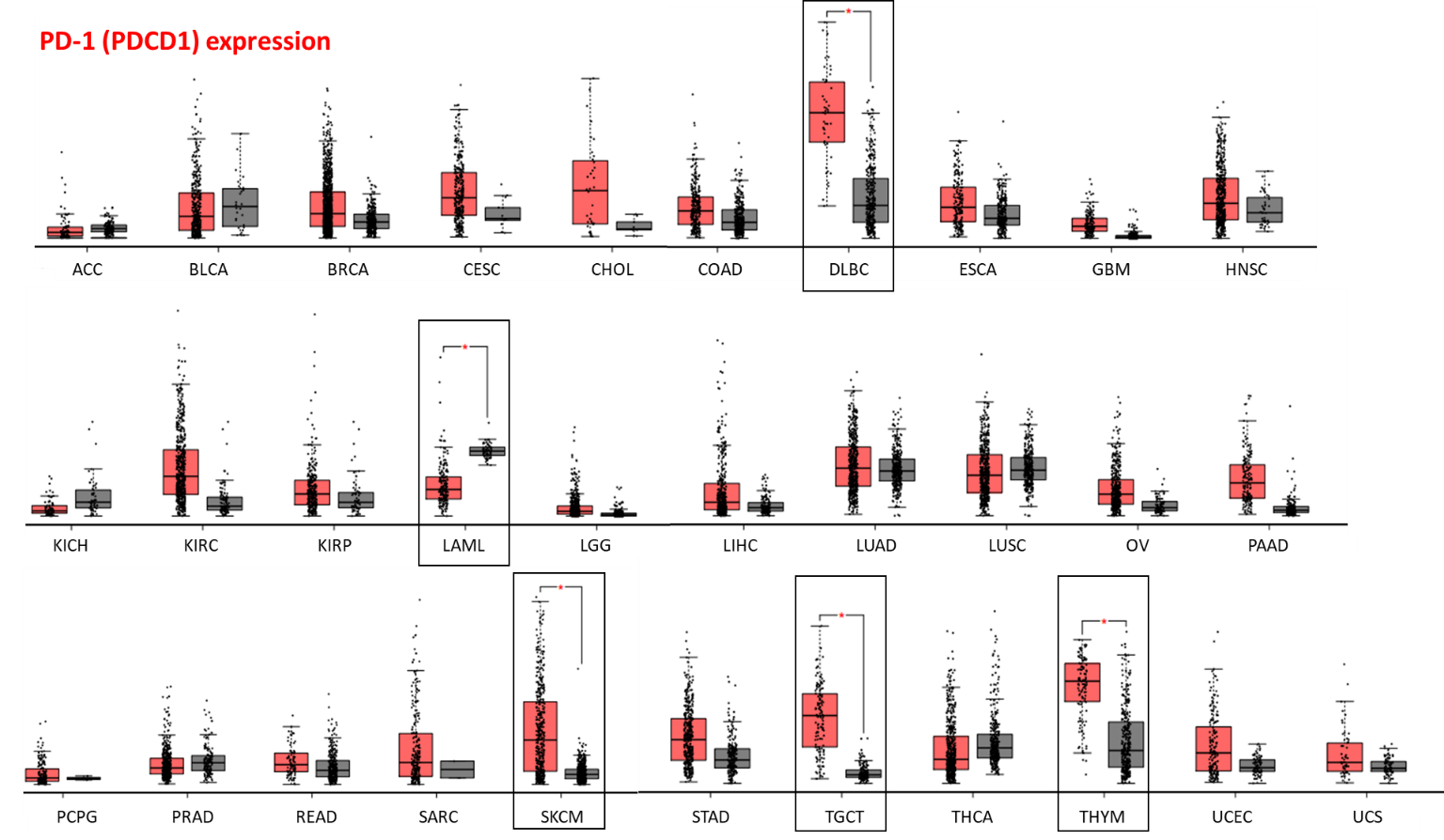


**Figure S2.** Gene expression level of PD-1 in 31 cancer types and in matched healthy controls. Asterisks indicate significant (p < 0.0001) difference.

**Figure S3**


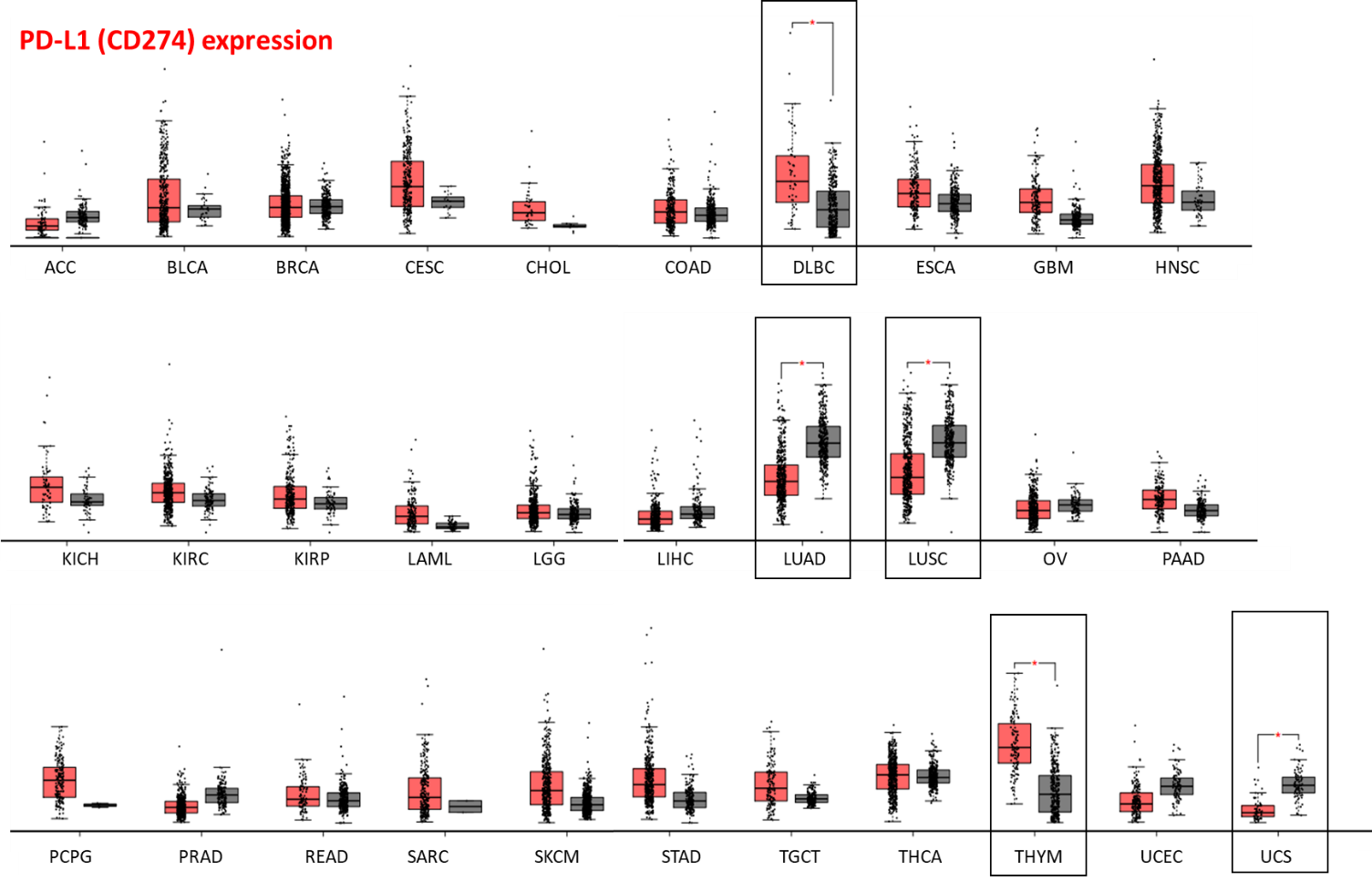


**Figure S3.**  Gene expression level of PD-L1 in 31 cancer types and in matched healthy controls. Asterisks indicate significant (p < 0.0001) difference.

**Figure S4**


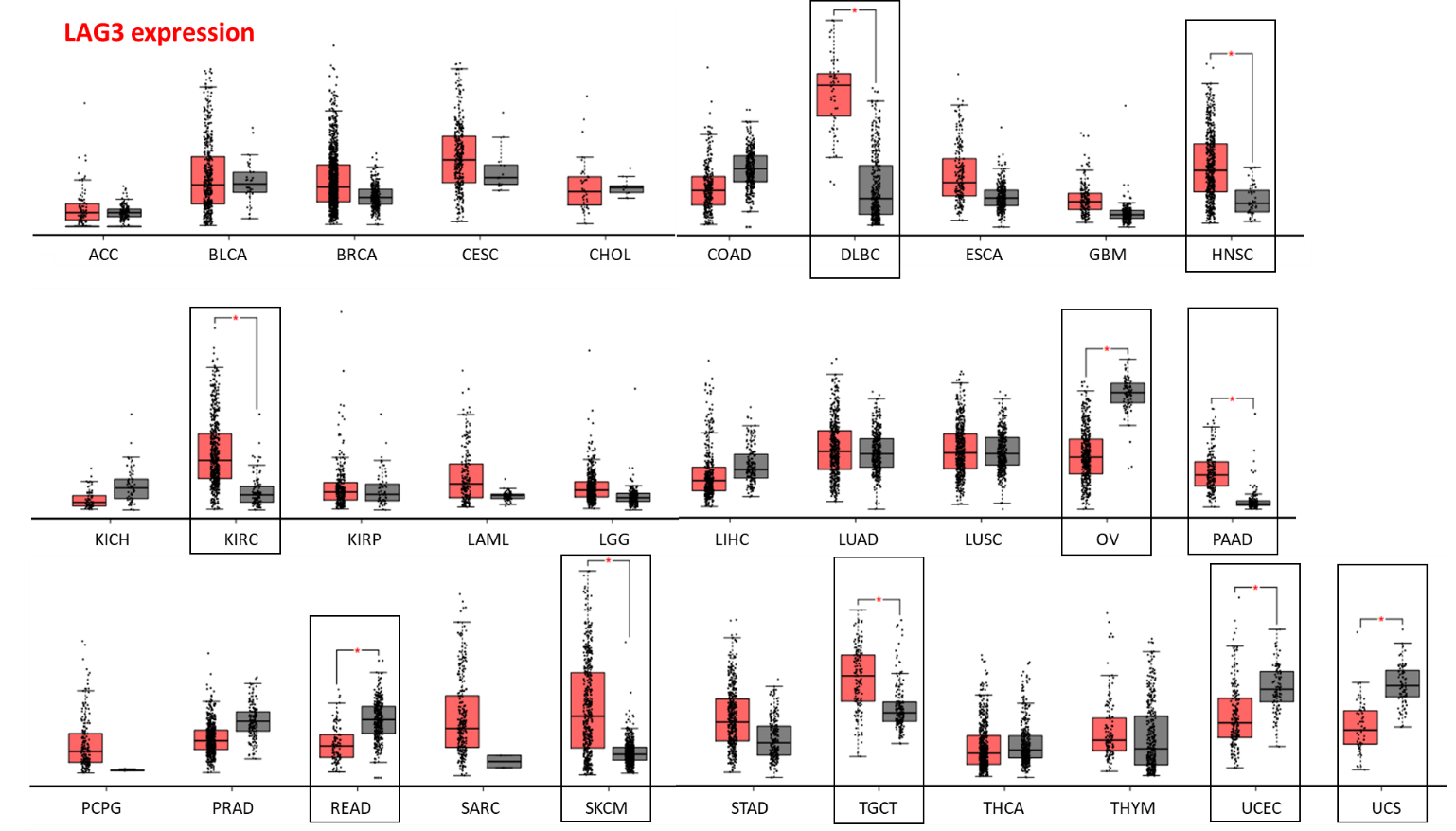


**Figure S4.** Gene expression level of LAG3 in 31 cancer types and in matched healthy controls. Asterisks indicate significant (p < 0.0001) difference.

**Figure S5**


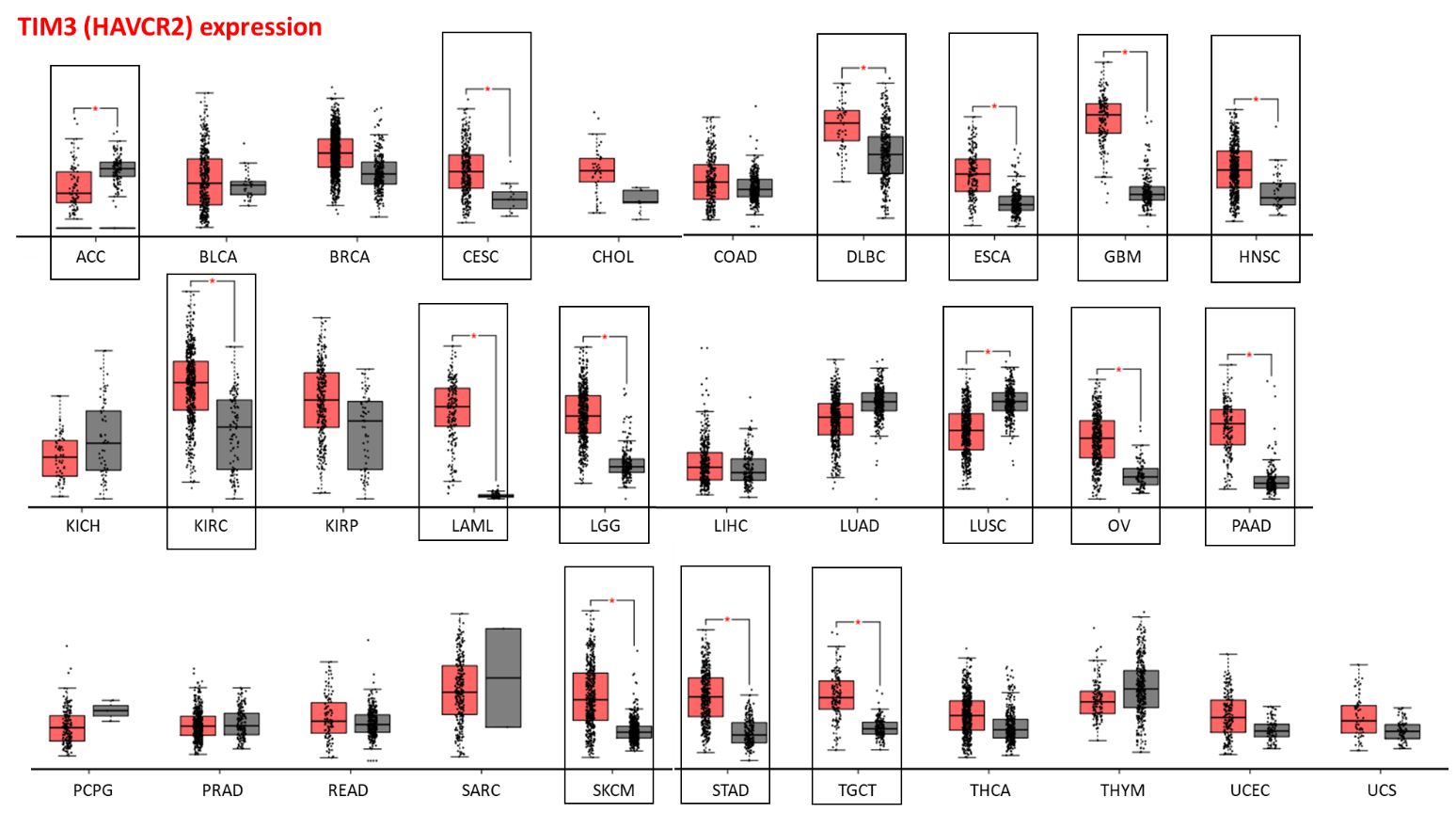


**Figure S5.** Gene expression level of TIM3 in 31 cancer types and in matched healthy controls. Asterisks indicate significant (p < 0.0001) difference.

**Figure S6**

**
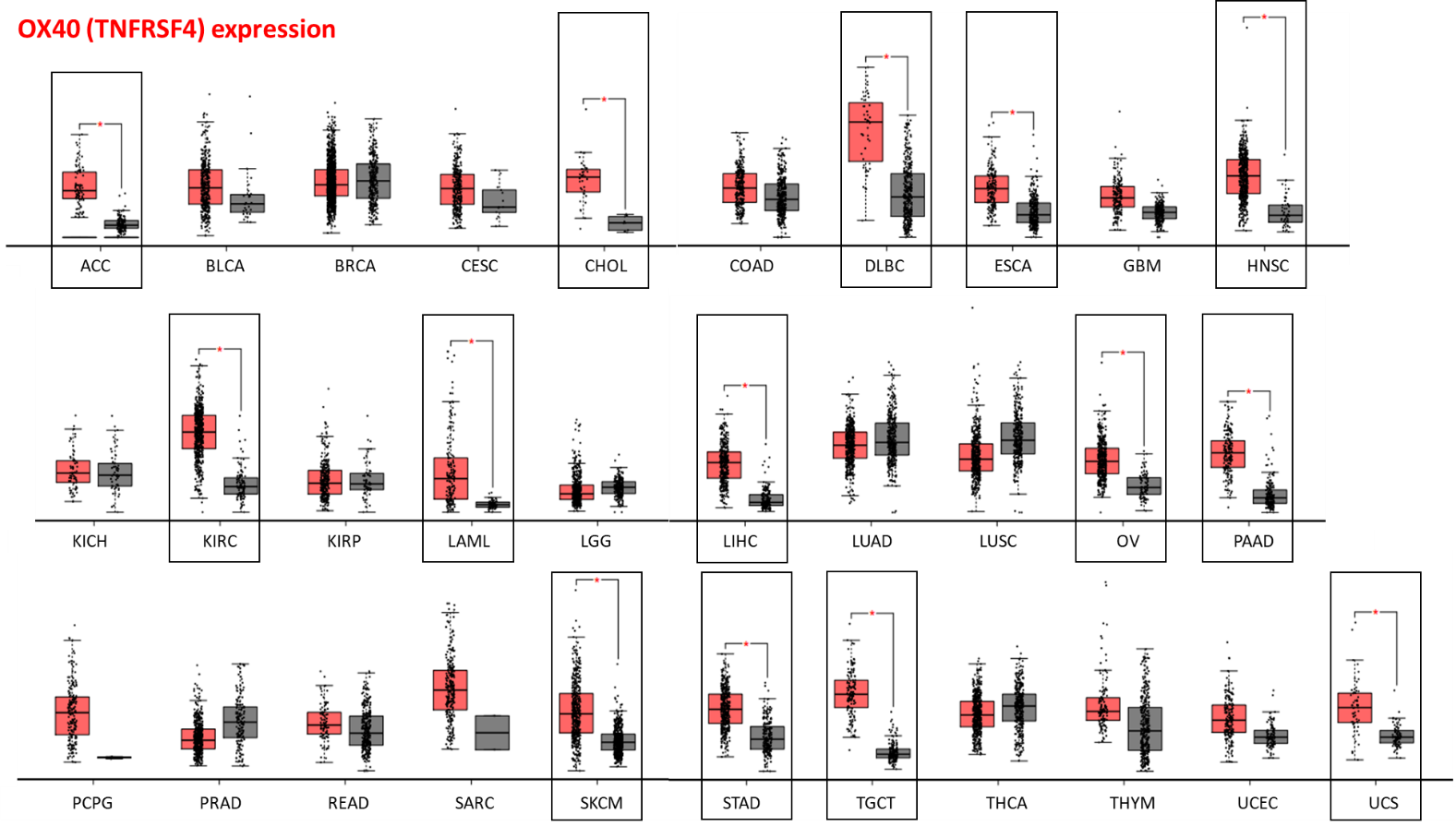
**

**Figure S6.** Gene expression level of OX40 in 31 cancer types and in matched healthy controls. Asterisks indicate significant (p < 0.0001) difference.

**Figure S7**


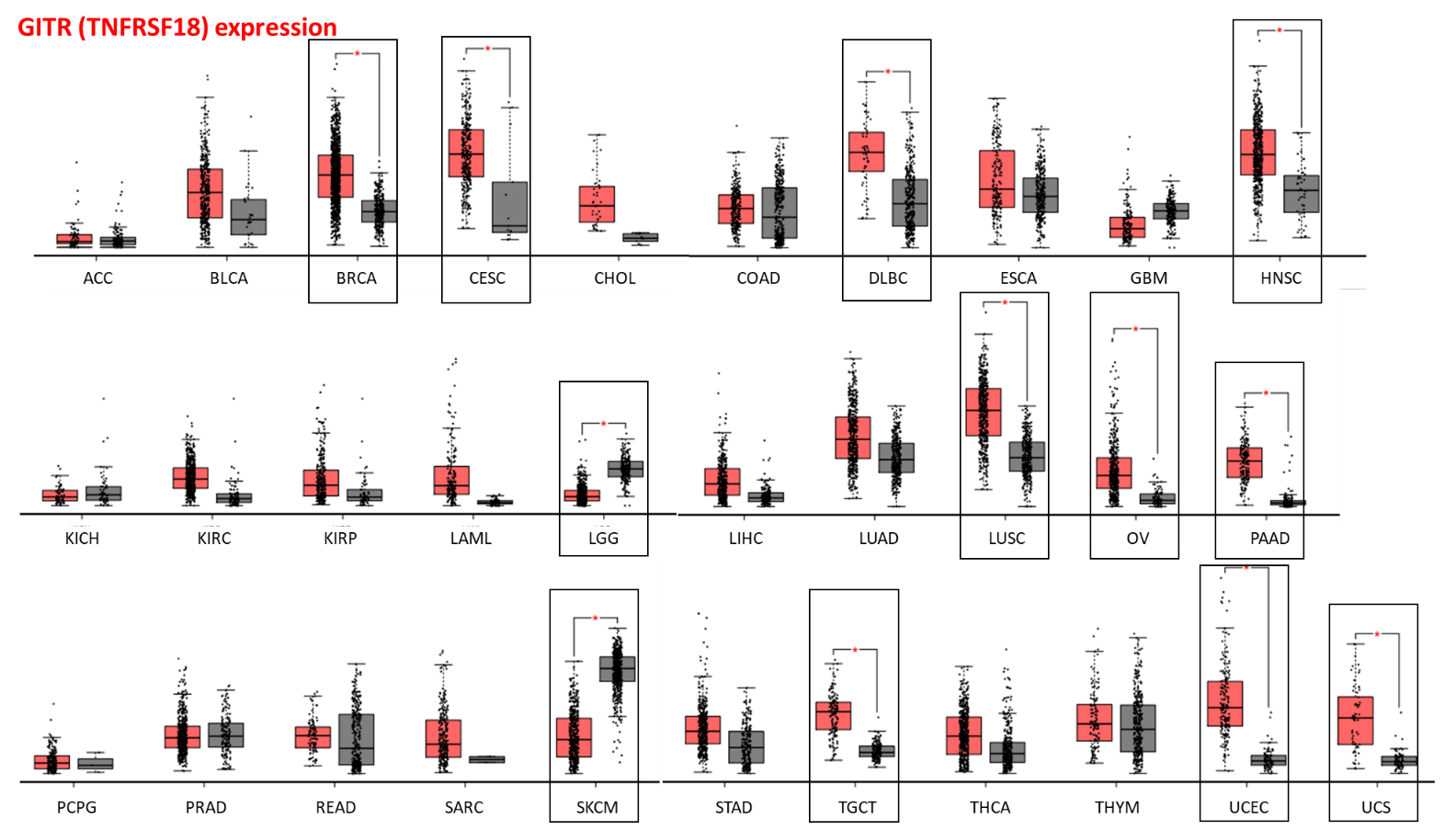


**Figure S7.** Gene expression level of GITR in 31 cancer types and in matched healthy controls. Asterisks indicate significant (p < 0.0001) difference.

**Figure S8**

**
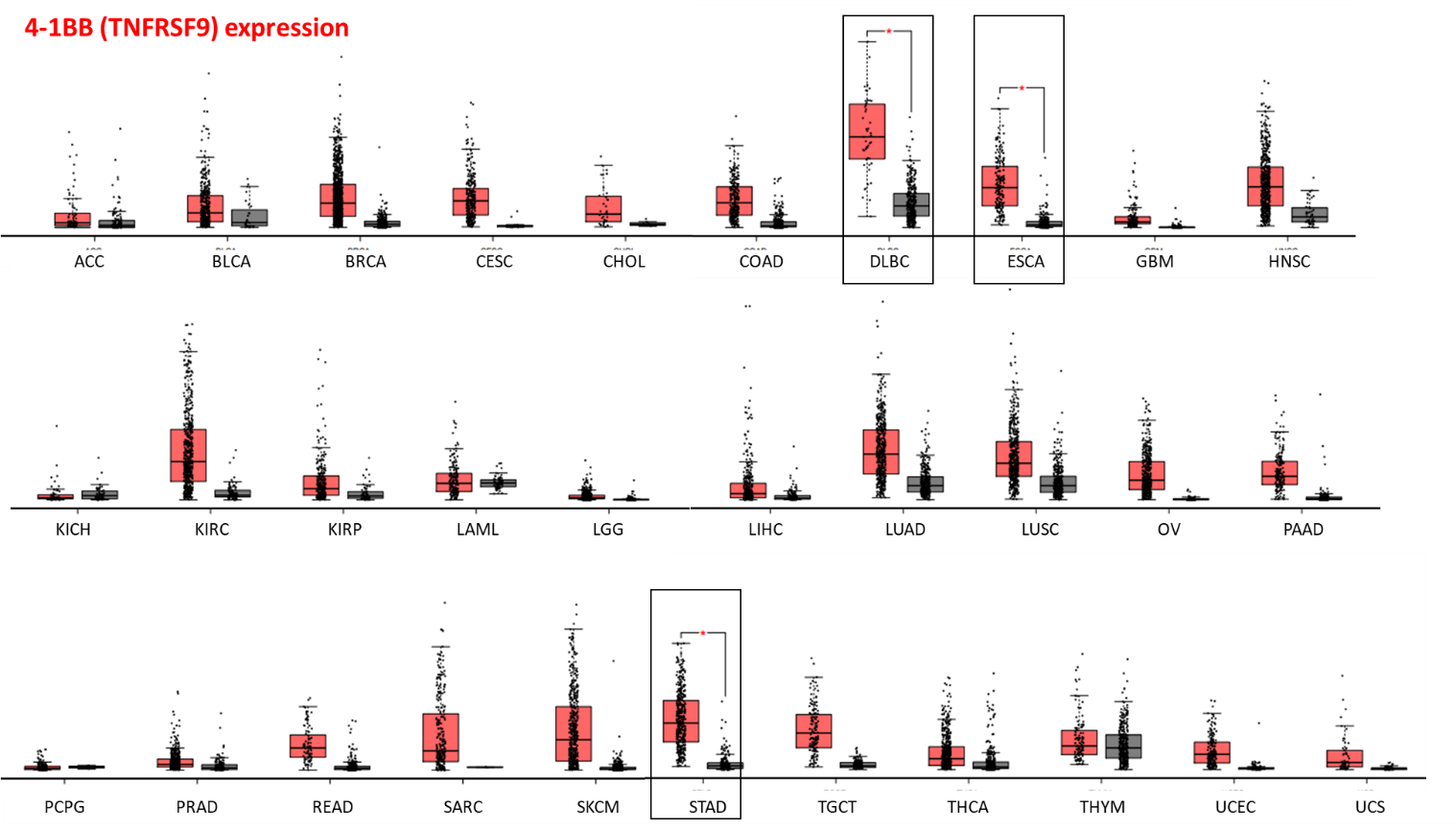
**

**Figure S8.** Gene expression level of 4-1BB in 31 cancer types and in matched healthy controls. Asterisks indicate significant (p < 0.0001) difference.

**Figure S9**


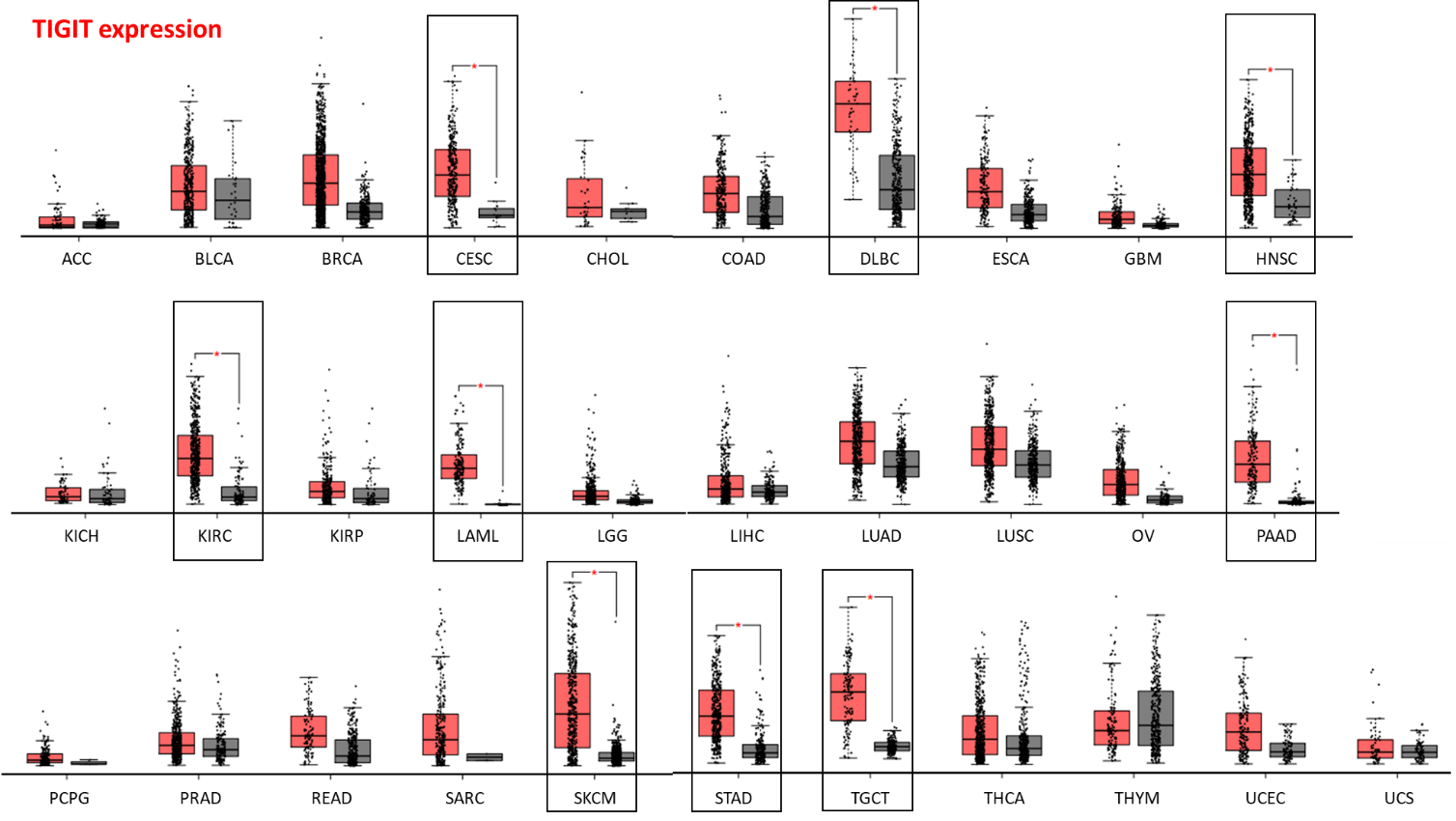


**Figure S9.** Gene expression level of TIGIT in 31 cancer types and in matched healthy controls. Asterisks indicate significant (p < 0.0001) difference.

**Not shown Table S1.**

Clinical trials reported in Clinicaltrials.gov referring to each ICR molecule investigated.

| **Molecule/target molecule** | **Clinical trials reported in Clinicaltrials.gov** |
| --- | --- |
| **CTLA4** | 665 |
| **PD-1 (PDCD1)** | 3226 |
| **PD-L1 (CD274)** | 2518 |
| **LAG3** | 121 |
| **TIM3 (HAVCR2)** | 100 |
| **OX40 (TNFRSF4)** | 78 |
| **GITR (TNFRSF18)** | 25 |
| **TIGIT** | 75 |
| **4-1BB (TNFRSF9)** | 124 |

**NOT Shown Table S2**.

Number of clinical trials using an immunotherapy-related approach

| **Search formula:**  ***“Cancer type” AND “immunotherapy”*** | **N. Clinical Trials reported** |
| --- | --- |
| Adernocortical cancer | 6 |
| Bladder cancer | 146 |
| Breast cancer | 355 |
| Cervical cancer | 166 |
| Cholangiocarcinoma | 55 |
| Colon carcinoma | 85 |
| Lymphoid neoplasm | 162 |
| Esophageal cancer | 151 |
| Gliobastoma | 159 |
| Head and neck cancer | 205 |
| Kidney cancer | 252 |
| Leukemia | 365 |
| Glioma | 207 |
| Liver cancer | 253 |
| Lung cancer | 927 |
| Ovary cancer | 187 |
| Pancreatic cancer | 197 |
| Pheochormocytoma + Paraganglioma | 4 |
| Prostate cancer | 202 |
| Rectum cancer | 64 |
| Sarcoma | 152 |
| Melanoma | 574 |
| Stomach cancer | 159 |
| Testis cancer | 18 |
| Thyroid | 22 |
| Thymoma | 5 |
| Uterine cancer | 94 |

**NOT Shown Table S3**.

**Validation of ICRs expression in EBI Expression atlas, in melanoma samples.** Up-regulation and down-regulation vs. controls, observed in EBI Expression atlas, reflects the regulation observed in GEPIA2 and reported in Table S2 and Figs S1 to S9.

| **Gene name** | **Expression level in melanoma** (36 biological replicates) (TPM) | **Expression level in normal skin, lower leg** (141 biological replicates) (TPM) | **Expression level in normal skin suprapubic** (43 biological replicates) (TPM) |
| --- | --- | --- | --- |
| TIM3 (HAVCR2) | 11 | 2 | 2 |
| OX40 (TNFRSF4) | 7 | 2 | 2 |
| GITR (TNFRSF18) | 2 | 33 | 38 |
| TIGIT | 2 | below cutoff | below cutoff |
| LAG3 | 4 | 1 | 2 |
| PD-L1 (CD274) | 2 | 1 | 1 |
| PD-1 (PDCD1) | 2 | 0.6 | 0.9 |
| CTLA4 | 3 | 0.6 | 0.9 |
| 4-1BB (TNFRSF9) | 2 | below cutoff | below cutoff |

**NOT Shown Table S4**.

**Validation of ICRs expression in EBI expression atlas, in pancreatic adenocarcinoma samples.** Up-regulation observed in EBI expression atlas, reflects the regulation observed in GEPIA2 and reported in Table S2 and Figs S1 to S9.

| **Gene name** | **Expression in pancreas adenocarcinoma** (75 biological replicates) (TPM) | **Expression in adjacent normal pancreas** (70 biological replicates) (TPM) |
| --- | --- | --- |
| TIM3 (HAVCR2) | 25 | 1 |
| OX40 (TNFRSF4) | 5 | 1 |
| GITR (TNFRSF18) | 4 | below cutoff |
| TIGIT | 3 | below cutoff |
| LAG3 | 2 | below cutoff |
| PD-L1 (CD274) | 6 | 2 |
| PD-1 (PDCD1) | 6 | below cutoff |
| CTLA4 | 4 | below cutoff |
| 4-1BB (TNFRSF9) | 4 | below cutoff |

**NOT Shown Table S5**.

**Validation of ICRs expression in EBI Expression atlas, in Thyroid carcinoma samples.** The absence of -or marginally- differential regulation, observed for most ICRs in EBI Expression atlas, reflects the absence of differential regulation observed in GEPIA2 reported in Table S2 and Figs S1 to S9.

| **Gene name** | **Expression in pancreas thyroid carcinoma** (47 biological replicates) (TPM) | **Expression in adjacent thyroid pancreas** (4 biological replicates) (TPM) |
| --- | --- | --- |
| TIM3 (HAVCR2) | 3 | 3 |
| OX40 (TNFRSF4) | 8 | 2 |
| GITR (TNFRSF18) | 3 | 2 |
| TIGIT | 0.8 | 3 |
| LAG3 | 1 | 3 |
| PD-L1 (CD274) | 4 | 5 |
| PD-1 (PDCD1) | 2 | 4 |
| CTLA4 | 0.8 | 2 |
| 4-1BB (TNFRSF9) | Below cutoff | 0.8 |
